# Running Performance of Soccer Players During Matches in the 2018 FIFA World Cup: Differences Among Confederations

**DOI:** 10.3389/fpsyg.2019.01044

**Published:** 2019-05-07

**Authors:** Quan Tuo, Lei Wang, Guohu Huang, Hengliang Zhang, Hongyou Liu

**Affiliations:** National Demonstration Center for Experimental Sports Science Education, South China Normal University, Guangzhou, China

**Keywords:** football, physical performance, match analysis, performance analysis, running distance

## Abstract

With the purpose of quantifying the differences in the running performance of soccer players during matches from different continental confederations, data of 1508 match observations generated from 559 players in 59 matches at the 2018 FIFA World Cup held in Russia were analyzed. Generalized mixed linear modeling was carried out to estimate the effect of confederations on each of the selected thirteen match running performance related variables (total distance covered, top speed achieved, number of sprints, distance covered and time spent in walking, jogging, low-speed running, moderate-speed running, and high-speed running), controlling the effects of match result, competition phase, and team and opponent strength. Results showed that the differences in the match running performance of UEFA and CONMEBOL players were trivial (ES between 0.04 and 0.14); players from AFC, CAF, and CONCACAF covered less total distance (ES between 0.26 and 0.54), spent less playing time, and covered less distance in jogging and low-speed running (ES between 0.20 and 0.53), whereas they spent more time walking (ES between 0.27 and 0.41) as compared with players from UEFA and CONMEBOL; top speed achieved, number of sprints made, and time spent and distance covered in the moderate- and high-speed running intensity zones by players from all confederations were similar (ES between 0.01 and 0.15), with an exception that high-speed-running distance covered by CONCACAF players was less than that by CAF players (2.0 ± 1.5 m/min vs. 2.3 ± 1.7 m/min, ES = 0.23, ±90% CL: ±0.21).

## Introduction

Being one of the most popular sports, soccer (association football) is believed to be a “universal language” (Sarmento et al., 2013). However, due to differences in the geographical, cultural, historical, and social aspects, soccer teams from different countries and continents are often characterized by different particularities of match-play (Sarmento et al., 2013; Sapp et al., 2018).

On the basis of a qualitative analysis, (Sarmento et al., 2013) discussed the differences in the preferred strategy and tactics, players’ characteristics, and coaches’ philosophy existing among the English Premier League, Italian *Serie A*, and Spanish *La Liga.* It was concluded that English Premier League teams prefer the direct playing style, Italian teams are characterized by their rigid tactical requirement and defensive organization, whereas Spanish teams prioritize the technical beauty of the game and prefer to control the game by passing.

Apart from that, many quantitative studies compared the differences between major European leagues in aspects of anthropometric data, refereeing, player recruitment, injury pattern, competitive balance, and technical and physical match performance. Bloomfield et al. (2005) found that, in the “big five” European soccer leagues (English Premier League, French *Ligue 1*, German *Bundesliga*, Italian *Serie A*, Spanish *La Liga*), players from the *Bundesliga* had the greatest stature, body mass, and BMI, while *La Liga* players had the shortest stature, while the *Serie A* players had the least body mass and BMI. Sapp et al. (2018) indicated that the English Premier League is the most aggressive league with the lowest number of yellow and red cards per match, supporting the notion of the most lenient refereeing in the “big five.” Littlewood et al. (2011) identified that teams from the *Bundesliga* and Premier League are the biggest recruiter of foreign players, while *La Liga* and *Serie A* teams host the most indigenous home-grown players; while *Ligue 1* teams have the most non-indigenous home-grown players. Waldén et al. (2005) showed that the risk of match injury was significantly higher in the English and Dutch teams than in the teams from France, Italy, and Spain. Pawlowski et al. (2010) demonstrated that Italian *Serie A* was the most unbalanced competition in the “big five,” whereas French *Ligue 1* was the most balanced competition during the 2001–2008 period. Oberstone (2011) investigated the technical match performance of the English Premier League, Italian *Serie A*, and Spanish *La Liga*, and found that teams from the English Premier League had a significantly lower percentage of shots on target than teams from the other two leagues, whereas *Serie A* teams had the highest percentage of successful tackles among the three leagues. Dellal et al. (2011) compared both the physical and technical match performance of soccer players in the English Premier League and Spanish *La Liga*. Their main findings included that the total distance covered by players from the English Premier League and *La Liga* was non-significant, but Premier League players generally covered greater distances in sprinting, while players from both leagues performed a similar proportion of successful passes.

The FIFA World Cup is the highest-level international soccer tournament contested by national teams throughout the world. It provides an ideal sample to study the match performance of soccer players from different continental confederations. Thus, the current study aims to analyze the running performance of soccer players during matches in the 2018 FIFA World Cup held in Russia to identify if there is a “continental difference” in the context of modern soccer.

## Materials and Methods

### Data Resource

Match data were retrieved from the official website of FIFA (FIFA, 2018). The original data were collected using a real-time optical tracking system operated at 25 frames per second that provided details of players’ activities on the field. The accuracy of the tracking system has been verified by the Centre for Football Research, Liverpool John Moores University^1^. The updated tracking system has been recently tested by Linke et al. (2018) and has an acceptable reliability. For previous applications of FIFA’s database, refer to the examples given by Da Mota et al. (2015) and Nassis et al. (2015). The research committee of the local university approved this study. The committee believed that the object investigated in this study was match-performance data generated by soccer players, which did not involve direct human or animal subjects; hence, no written informed consent was required.

### Sample and Variables

There were thirty-two teams from five different continental confederations that participated in the sixty-four matches of the 2018 FIFA World Cup. These five confederations are: Asian Football Confederation (AFC, five teams), Confederation of African Football (CAF, five teams), Confederation of North, Central American and Caribbean Association Football (CONCACAF, three teams), Confederación Sudamericana de Fútbol (CONMEBOL, 5 teams) and the Union of European Football Associations (UEFA, fourteen teams). In line with Da Mota et al. (2015), we selected fifty-nine of sixty-four matches from the tournament for our analysis, excluding the five matches in which extra time was played. Observations generated from all out-field players who participated in these fifty-nine matches were selected as our sample (including the full-match, substituted-on, and substituted-out players). Match data of goalkeepers were excluded because of the specificity of this position. The final sample included 1508 match observations generated by 559 players (see Table 1 for more details). On the basis of the availability of data, thirteen variables were selected to quantify the running performance of players during matches. Detailed operational definitions of these variables can be found in Table 2. The running intensity intervals were set by the FIFA official tracking system (FIFA, 2018). Referencing the intervals used by Da Mota et al. (2015) and Nassis et al. (2015), we classified the intensity zones into the following: zone 1, walking; zone 2, jogging; zone 3, low-speed running; zone 4, moderate-speed running; zone 5, high-speed running.

**TABLE 1:** Detailed sample distribution.

	UEFA	CONMEBOL	AFC	CAF	CONCACAF	Total
Number of players	250	87	82	83	57	559
Age^#^ (mean ± SD)	27.5 ± 3.8	28.1 ± 3.6	27.9 ± 3.4	26.8 ± 3.6	28.9 ± 4.5	27.7 ± 3.8
Caps* (mean ± SD)	41.6 ± 31.6	42.9 ± 33.6	42.2 ± 32.4	29.2 ± 21.6	61.6 ± 37.7	42.0 ± 32.3
Match observations (min-max)	730 (1–7)	259 (1–5)	194 (1–4)	194 (1–3)	131 (1–4)	1508 (1–7)

^#^Age on 14th June 2018 (opening day of the 2018 FIFA World Cup) calculated from the birth dates of players. *Number of matches played for the national team. The information of birth date and caps was derived from the “FWC_2018_Squadlists” published on the FIFA official website.

**TABLE 2:** Selected dependent variables.

Variable (unit)	Definition
Total Distance (m⋅min^–1^)	Total distance covered by a player during match play relativized to per minute
Top Speed (km/h)	Maximum running velocity of a player during match play
Sprint (times⋅min^–1^)	Efforts of sprint (velocity >20 km/h) achieved by a player during match play relativized to per minute
Time zone 1 (%)	Percentage of time moving at the velocity of 0–7 km/h out of total playing time
Time zone 2 (%)	Percentage of time moving at the velocity of 7–15 km/h out of total playing time
Time zone 3 (%)	Percentage of time moving at the velocity of 15–20 km/h out of total playing time
Time zone 4 (%)	Percentage of time moving at the velocity of 20–25 km/h out of total playing time
Time zone 5 (%)	Percentage of time moving at the velocity of >25 km/h out of total playing time
Distance zone1 (m⋅min^–1^)	Distance covered at the velocity of 0–7 km/h by a player during match play relativized to per minute
Distance zone2 (m⋅min^–1^)	Distance covered at the velocity of 7–15 km/h by a player during match play relativized to per minute
Distance zone3 (m⋅min^–1^)	Distance covered at the velocity of 15–20 km/h by a player during match play relativized to per minute
Distance zone4 (m⋅min^–1^)	Distance covered at the velocity of 20–25 km/h by a player during match play relativized to per minute
Distance zone5 (m⋅min^–1^)	Distance covered at the velocity of >25 km/h by a player during match play relativized to per minute

## Procedure and Statistical Analysis

Generalized mixed linear modeling was realized with *Proc Glimmix* in the University Edition of Statistical Analysis System (version SAS Studio 3.6). The confederation variables, match result, competition phase, and team and opponent strength were included in the modeling as the fixed effects. Random effects for player name, team identity, and match identity were added to account for repeated measurement on the players, teams, and matches. Separate Poisson regressions were run in the modeling, taking the value of each of the thirteen match running performance-related variables as dependent variables.

Confederation, match result, and competition phase were all included as nominal predictor variables in the model. Confederation was with five levels (UEFA, CONMEBOL, AFC, CAF, and CONCACAF), match result was with three levels (win, draw, and loss), and competition phase was with two levels (group stage and knock-out stage). The effect of team and opponent strength was estimated by including the difference in the log of the FIFA Ranking as a predictor (Yi et al., 2018). By adding the fixed effects of match result, competition phase, and team and opponent strength, the pure differences among confederations can be more accurately assessed.

Uncertainty in the true effects of the predictors was evaluated using non-clinical magnitude-based inferences as implemented in the spreadsheet accompanying the package of materials for generalized mixed modeling with SAS Studio (Hopkins, 2016). Estimated magnitudes and their confidence limits were expressed in standardized units, and were assessed qualitatively with the following scale: <0.2 trivial, 0.2–0.6 small, 0.6–1.2 moderate, 1.2–2.0 large, >2.0 very large (Hopkins et al., 2009). Standardization was achieved by dividing the estimated effect by the between-player standard deviation, which was derived from the mixed model by adding the variance for the true differences between players to the team-to-team and match-to-match variance within players before taking the square root. Effects were deemed clear if the 90% confidence interval did not include substantial positive and negative values simultaneously. Clear effects were reported with a qualitative likelihood that the true effect was either substantial or trivial (whichever probability was greater) using the following scale: <0.5% most unlikely, 0.5–5% very unlikely, 5–25% unlikely, 25–75% possibly, 75–95% likely, 95–99.5% very likely, >99.5% most likely (Hopkins et al., 2009).

## Results

**FIGURE 1:**
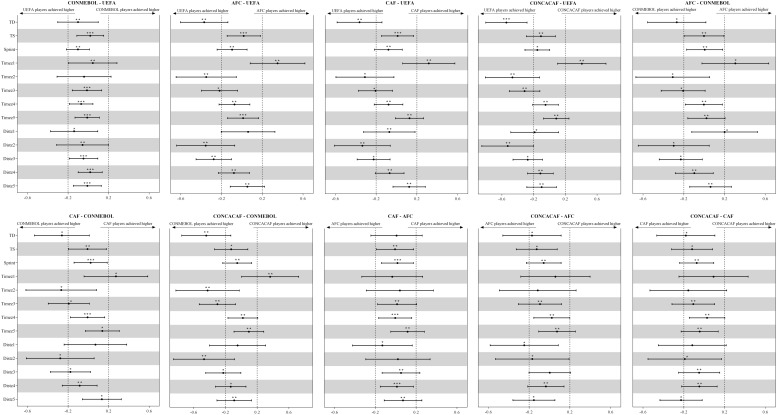
Standardized differences among running performance of players from different continental confederations. Bars are 90% confidence intervals. Asterisks indicate the likelihood for the magnitude of the true difference as follows: *possible; **likely; ***very likely. Asterisks located in the area between –0.2 and 0.2 denote for trivial differences.

Descriptive statistics and differences in the running performances of the players during matches from different continental confederations estimated from the generalized mixed linear modeling are presented in Table 3 and Figure 1.

**TABLE 3:** Descriptive statistics of the match running performance of soccer players in the 2018 FIFA World Cup estimated from the generalized mixed linear model.

Variable	UEFA	CONMEBOL	AFC	CAF	CONCACAF
TD	107±12	105±12	102±11	102±11	100±11
Top speed	28.1±2.8	28.2±2.8	28.2±2.8	28.2±2.8	27.8±2.7
Sprint	0.36±0.16	0.35±0.15	0.35±0.15	0.35±0.15	0.34±0.15
Time zone 1	66.3±6.0	66.5±6.0	68.4±6.2	68.2±6.2	68.8±6.2
Time zone 2	25.4±4.6	25.3±4.6	23.9±4.4	24.1±4.4	23.4±4.3
Time zone 3	5.4±1.8	5.4±1.8	5.0±1.7	5.1±1.7	4.9±1.7
Time zone 4	1.69±0.77	1.64±0.75	1.64±0.75	1.64±0.75	1.66±0.76
Time zone 5	0.32±0.67	0.32±0.66	0.33±0.67	0.39±0.74	0.36±0.72
Distance zone 1	37.7±3.8	37.1±3.7	37.9±3.8	37.4±3.8	37.0±3.7
Distance zone 2	44.5±8.3	44.0±8.2	41.6±7.8	41.8±7.9	40.3±7.6
Distance zone 3	15.1±4.9	14.9±4.9	13.8±4.6	14.1±4.6	13.9±4.6
Distance zone 4	6.1±2.5	6.1±2.5	5.9±2.4	5.9±2.5	5.8±2.4
Distance zone 5	2.1±1.6	2.1±1.6	2.2±1.6	2.3±1.7	2.0±1.5

The match running performances of UEFA and CONMEBOL players were similar. The total distance covered by AFC, CAF, and CONCACAF players were substantially less than that covered UEFA and CONMEBOL players to a small magnitude. Players from AFC, CAF, and CONCACAF spent substantially more time walking but less time jogging and low-speed running than did players from UEFA and CONMEBOL. Consequently, AFC, CAF, and CONCACAF players covered less distance in jogging and low-speed running than UEFA and CONMEBOL players. The differences in the top speed achieved, number of sprints made, time spent and distance covered in the moderate- and high-speed running intensity zones by players from all confederations were clearly trivial, with the exception that the high-speed-running distance covered by CONCACAF players was substantially less than that covered by CAF players.

## Discussion

The current study quantified the differences in the running performance of soccer players during matches from different continental confederations participating in the 2018 FIFA World Cup held in Russia. Our main results showed that the difference in the running performance of UEFA and CONMEBOL players were trivial; players from AFC, CAF, and CONCACAF covered less total distance, spent less playing time and covered less distance in jogging and low-speed running, while they spent more time in walking than players from UEFA and CONMEBOL; top speed achieved, number of sprints made, time spent and distance covered in the moderate- and high-speed running intensity zones by players from all confederations were similar, with an exception that high-speed-running distance covered by CONCACAF players was less than CAF players.

It has been generally accepted that soccer players and teams from different leagues, countries, and confederations are always attributed to different characteristics (Crolley et al., 2000; Wong, 2008). Previous studies investigating these differences mainly focused on comparing the European domestic leagues. Major results indicated that soccer teams from the English Premier League prefer the direct playing style with a special emphasis on the physical aspect, Italian teams are especially characterized by their rigid tactical requirement and defensive organization, while Spanish teams prioritize the technical beauty of the game and players have better ability to control the game (Oberstone, 2011; Sarmento et al., 2013; Sapp et al., 2018). Dellal et al. (2011) analyzed the running performance of soccer players during matches from the English Premier League and Spanish *La Liga*, and concluded that there was no overall difference between these two leagues in total distance covered. However, not much is known about the differences in soccer players among different continental confederations (Wong, 2008), especially when it comes to the running performance during matches. An early study (Rienzi et al., 2000) reported that the total distance covered in matches by professional soccer players from South America was about 1500 m (8638 m vs. 10104 m) less than that of players from the English Premier League. While it contrasts with the result of Barros et al. (2007) who revealed that the mean total distance covered (10012 m) by Brazilian First Division soccer players was similar to European soccer players. Our results are in accordance with Dellal et al. (2011) and Barros et al. (2007), the total distance covered, as well as distance covered and match time spent in different speed zones, by UEFA players are analogous to CONMEBOL players.

High-intensity running is one of the most crucial elements in elite soccer match performance (Di Salvo et al., 2009; Dellal et al., 2011). The comparative analysis of Dellal et al. (2011) revealed that English Premier League players performed a substantially greater distance in high-intensity running than *La Liga* players. Nevertheless, our results showed that top speed achieved, number of sprints made, and time spent and distance covered in the moderate- and high-speed running intensity zones by players from nearly all confederations were similar in matches at the 2018 FIFA World Cup in Russia. Previous research on the 2010 and 2014 FIFA World Cup showed that players competing in this type of tournament may adopt a “pacing strategy” by modifying their match behaviors to maintain their ability to execute technical actions at a high level and to preserve their capacity to achieve high intensity activities by generating the peak running speeds (Nassis, 2013; Nassis et al., 2015; Chmura et al., 2017). One of the most common pacing strategies is that players spare low-intensity activities such as walking and jogging so as to preserve essential high-intensity running (Bradley and Noakes, 2013). According to our results which showed that AFC, CAF, and CONCACAF players spent more playing time walking, while they spent less time and covered less distance in jogging and low-speed running than players from UEFA and CONMEBOL, we can find that there was a difference in the pacing strategies executed by players from UEFA and CONMEBOL and players from the other three confederations. AFC, CAF, and CONCACAF players had to spare their jogging and low-speed running time and distance to guarantee their high intensity running activities, while UEFA and CONMENBOL players were able to achieve the same by saving their playing time by walking. Considering the truth that UEFA and CONMENBOL teams were superior in terms of FIFA rankings and the fact that teams from these two confederations took 14 out of the 16 qualified positions in the FIFA, 2018 World Cup, players of UEFA and CONMENBOL can be attributed to having the highest quality (Collet, 2013). Hence, the results from this study could reveal a trend that all elite soccer players can preserve that capacity to accomplish the essential high intensity activities reaching their top running speeds in different match contexts, while the best players can achieve this by recovering from jogging and low-speed running rather than from walking.

Some attention should be paid to the special case that the high-speed-running distance covered by CONCACAF players was less than that by CAF players. This finding may partially be explained by the difference in the age of players from these two confederations. The information of squads of all the teams in the 2018 World Cup (FIFA, 2018) showed that CONCACAF players who appeared in matches of this World Cup were the oldest (28.9 years on average) and the most experienced (61.6 national team caps on average), while the appeared CAF players were the youngest (26.8 years on average) and with the least experience (29.2 national team caps on average). According to Wong (2008), this was also the situation in the 2002 and 2006 editions of the FIFA World Cup. Wong (2008) further pointed out that the young squads of CAF may be due to the fact that the playing style in Africa was high-speed and high-intensity characterized, where younger players could normally perform better than older players.

### Practical Applications and Limitations

Results of this study could provide some practical information to soccer practitioners in several ways. On one hand, UEFA clubs that are used to recruiting elite soccer players from CONMEBOL domestic leagues could plan training drills for these newly recruited players, focusing more on the technical and tactical aspects rather than the physical ones, because the match running performance of elite players from these two confederations was analogous. On the other hand, training of elite soccer players should not only emphasize their ability to maintain high intensity activities but should also underline their capacity of recovery from jogging and low-speed running rather than from walking. Furthermore, when a relatively older squad is in hand, average age up to 29 years for example, coaches should be aware that the players may have some limitations to achieving high-speed running activities during the match; thus, a special training plan and match preparations are warranted.

There are some limitations in our study that should be further investigated. Firstly, the well-defined positional difference in the match running characteristics of players should be considered for future analysis. Secondly, the differences in the tactical and technical match performance of players from different confederations were not measured in this study, which are suggested to be combined. Lastly, due to the limitation in the accessibility of data, the sample distribution in this study is not homogeneous; moreover, the sample does not include the matches with extra time, neither does it exclude the matches with red cards. These shortcomings may lead to some bias in the results, which should be avoided in future research.

## Author Contributions

QT participated in the study design, statistical analysis, data interpretation, and manuscript preparation. LW, GH, and HZ achieved the literature search, data collection, and statistical analysis. HL managed the whole process of study design, literature search, data collection, statistical analysis, data interpretation, and manuscript preparation.

## Conflict of Interest Statement

The authors declare that the research was conducted in the absence of any commercial or financial relationships that could be construed as a potential conflict of interest.

## Footnotes

1 www.uefa.com
